# Dr. John Quincy Byram

**Published:** 1911-07-15

**Authors:** 


Dr. John Quincy Byram, of Indianapolis. It will be sad news for many dentists to learn of the sudden death of Dr. Byram. He was found hanging in the doorway of his office, by two of his dental friends, the night of June 28th. It is supposed that he had become despondent from a long period of ill health and the fear that he should loose his mental faculties, caused him to take his life. He has been suffering from a nervous breakdown for nearly two years and had done everything he could to regain his health. He gave up his college work and went in private practice and then to get out of doors engaged in real estate deals which were quite profitable financially, but his health gradually gave way and he felt that he could not longer endure the strain. Dr. Byram was an indefatigable worker and had high ideals and great ambitions and a temperment that would not allow him to rest or take things quietly.

Dr. Byram was a graduate of the Indiana Dental College and a teacher in that school for many years. Most of this time he gave all his time to teaching, but for the past three years he only lectured. He very early took a great interest in the work of the Institute of Dental Pedagogics and was always in attendance adding much to the programme by his contributions and discussion. He contributed most ably to the technical work of porcelain; his study of the color problem being the ablest work he ever produced. He took a very decided stand against the type of cavity formation first suggested for the porcelain inlay, and stood strenuously for a form of cavity preparation that would contain the principles of mechanical retention in addition to the cement adhesion.

He was a great worker in his local and state societies, and frequently contributed to other state and the national societies, by papers and clinics; always with such earnestness and enthusiasm as to carry conviction. He was a thorough professional gentleman and loved his profession above everything else and never tired in serving where he could.

He was an ardent member of the Delta Sigma Delta Fraternity and held all the positions of honor and trust in that organization, many loyal "Delta Sigs" will be grieved to learn of his untimely death, and there will be sorrow at the annual meeting this month in Cleveland.

Dr. Byram leaves a wife, a son twelve years of age, and a daughter seven years old. His family will have the cordial sympathy of a large number of dentists in all parts of the Country.

